# Cystic struma ovarii: a rare presentation of an infrequent tumor

**DOI:** 10.1590/S1516-31802000000100005

**Published:** 2000-01-02

**Authors:** Rita Barbosa de Carvalho, Maria Letícia Cintra, Patrícia Sabino de Matos, Paulo Sérgio Bueno de Campos

**Keywords:** Cystic struma ovarii, Frozen section, Teratoma, Struma ovarii, Exame de Congelação, Teratoma

## Abstract

**CONTEXT:**

Struma ovarii, a rare neoplasm, is a monophyletic teratoma composed of thyroid tissue. It is generally considered to account for less than 5% of mature teratomas.

**CASE REPORT:**

A diagnosis of struma ovarii may be the source of many diagnostic problems. It may be cystic and microscopic examination may only reveal a few typical thyroid follicles, resulting in confusion with other cystic ovarian tumors. Extensive sampling should be undertaken and immunohistochemistry may be decisive in establishing the thyroid nature of the epithelial lining. The authors report two cases of cystic struma ovarii, and discuss diagnostic criteria and the limitations of frozen biopsies in these tumors.

## resumo

**CONTEXTO:**

O diagnóstico de struma ovarii pode ser a fonte de muitos problemas diagnósticos. Pode ser cístico e o exame microscópico revelar somente poucos folículos típicos da tireóide, tendo por resultado a confusão com outros tumores ovarianos císticos. Uma amostragem extensiva deve ser realizada e imunohistoquímica pode ser decisiva para estabelecer a natureza do epitélio de revestimento.

**RELATO DE CASO:**

Os autores relatam dois casos do struma ovarii cístico e discutem critérios diagnósticos e limitações das biópsias de congelação nesses tumores.

## INTRODUCTION

Struma ovarii, a rare neoplasm, is a monophyletic teratoma composed of thyroid tissue. It is generally considered to account for less than 5% of mature teratomas. Its intraoperative evaluation can be difficult and errors in judging frozen sections may lead to overtreatment of patients.^1^ We recently encountered two cases of cystic struma that were misdiagnosed on frozen section. It was possible to make the definitive diagnosis only after examination of multiple sections on permanent material. The aim of the present study is to discuss diagnostic criteria and limitations of frozen biopsies in these tumors.

## CASE REPORT

### Case 1

**Figure 1:**
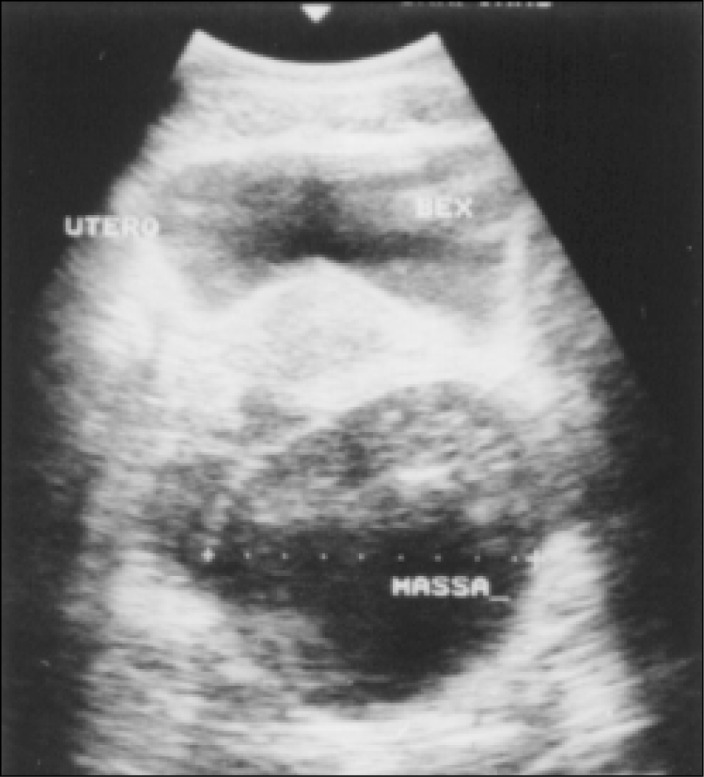
Case 1, adnexal cystic mass seen on ultrasound.

**Figure 2:**
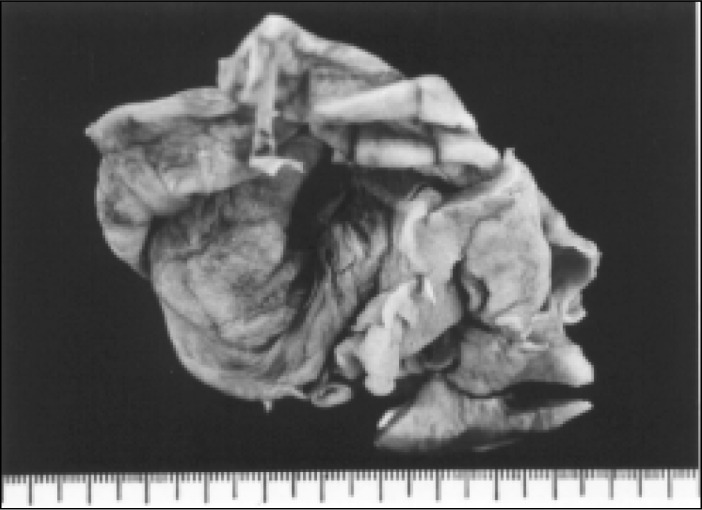
Case 1, predominantly unilocular cyst.

**Figure 3:**
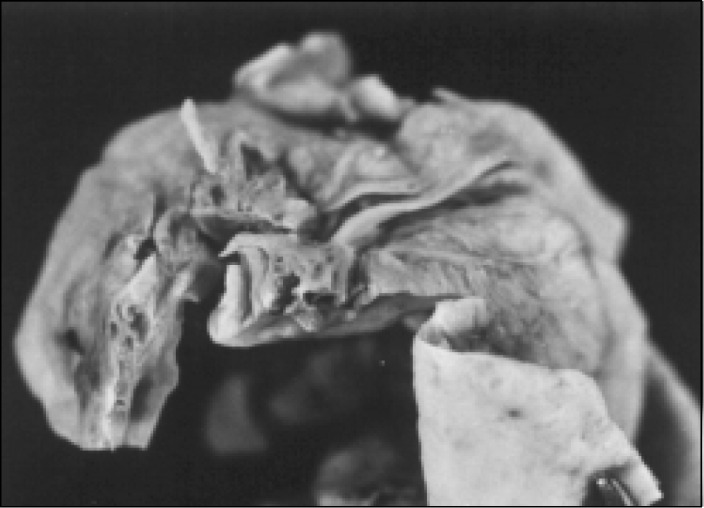
Case 1, wall of the cystic neoplasm presenting a microcystic area.

**Figure 4:**
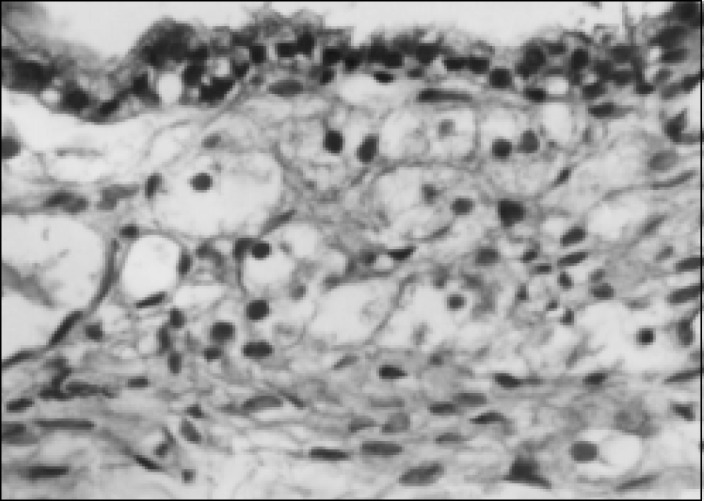
Case 1, cyst lined by nonspecific cuboid epithelium. Some foamy histiocytes are seen beneath the epithelium (Hematoxylin-eosin, 82X).

**Figure 5:**
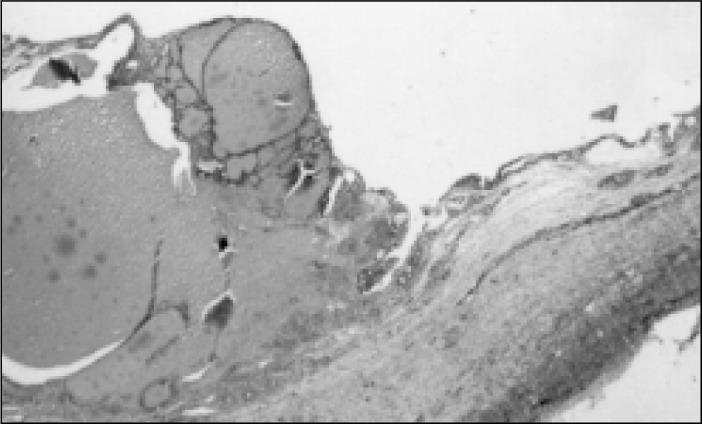
Case 1, aggregates of dilated thyroid follicles seen within the area shown in Figure 3 (Hematoxylin-eosin, 20X).

A 44-year-old white woman complaining of abdominal pain. The ultrasound (Fig. 1) showed a left adnexal cystic mass and exploratory laparotomy disclosed a cystic tumor in the left ovary. On frozen section examination it was classified as benign, and the histogenetic type as serous cystadenoma. The cyst (Fig. 2) was predominantly unilocular, 8cm in largest diameter. The wall and septa were thin, except for a 7x3cm microcystic gray area (Fig. 3) of thickness 0.5cm. Three sections were sent for frozen evaluation and 19 for embedding in paraffin blocks. The fibrous wall and septa were lined by epithelium of non-specific appearance (Figures 4 and 5). They were positive when stained for thyroglobulin and negative for vimentin, CEA and ID5 (estrogen receptor). Aggregates of dilated thyroid follicles were identified only within the area described above, in one single slide out of 19 examined.

### Case 2

**Figure 6:**
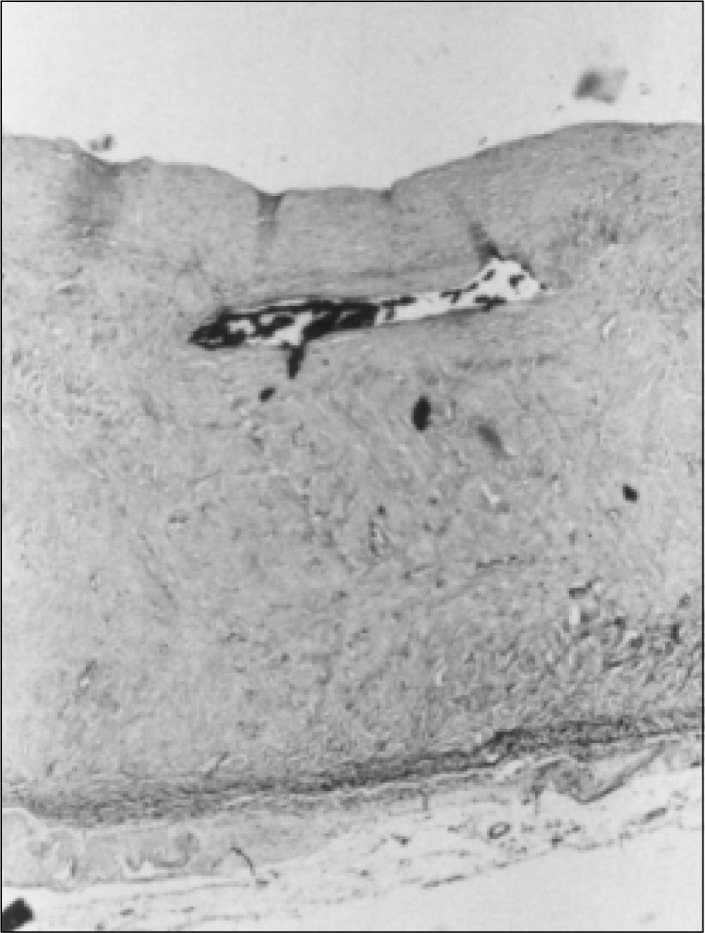
Case 2, calcified cystic fibrous wall and denuded epithelium (Hematoxylin-eosin, 41X).

**Figure 7:**
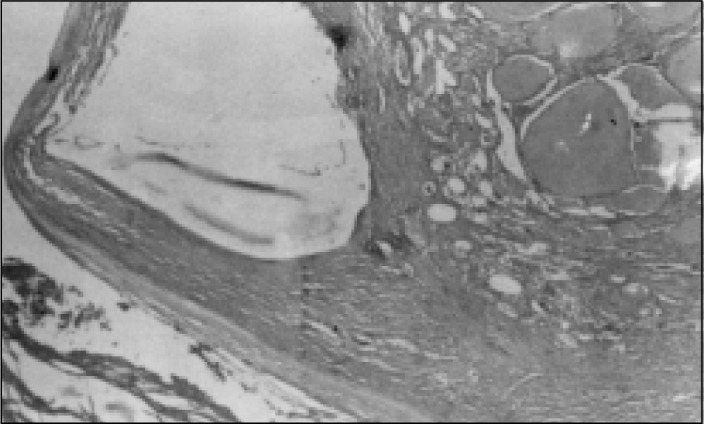
Case 2, collection of thyroid tissue within the cystic wall. (Hematoxylin-eosin, 41X).

A 60-year-old woman complaining of pelvic pain. On evaluation, she had a tender right adnexal mass. Computerized tomography scans demonstrated a multilocular mass, consistent with an ovarian tumor. She was subjected to exploratory laparotomy, in which a right adnexal cyst was found. The pathologist performed gross examination and observed extensive calcification of the wall. At the patient's discretion, only gross inspection was performed, and the diagnosis from this was benign, probably being a mature cystic teratoma. Selected portions of the tissues were well fixed and processed under decalcifying solutions. The walls and septa (Figures 6 and 7) were composed of extensively calcified fibrous tissue and lined by flat and frequently denuded epithelium. Scattered collections of thyroid follicles were seen within the septa in some sections.

### Ultrastructural features

**Figure 8:**
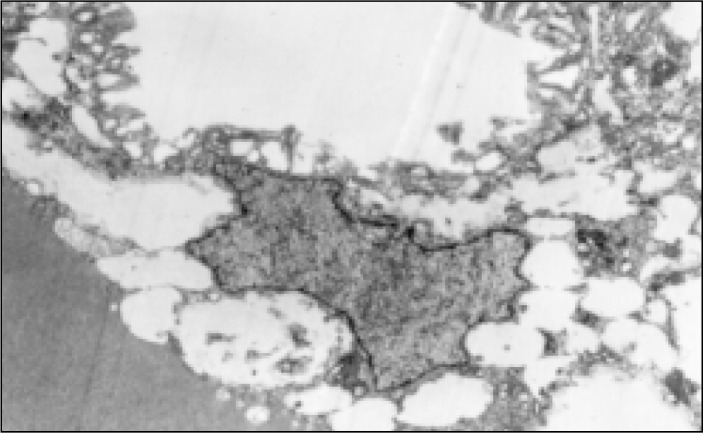
Case 1, epithelial cell showing microvilli on the apical side (Electron micrograph 7000 X).

**Figure 9:**
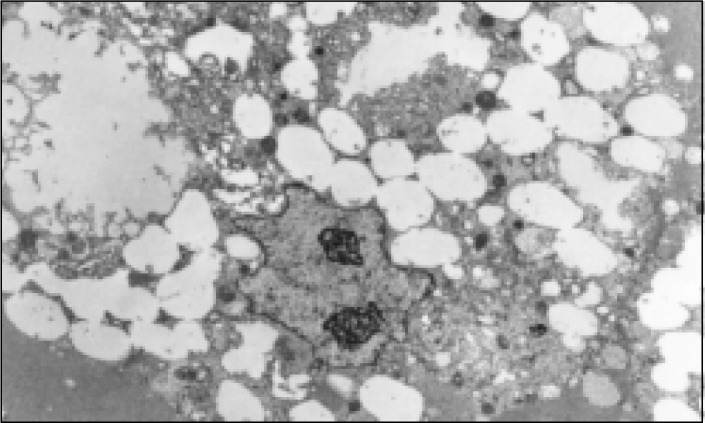
Case1, macrophages under the epithelium with intracellular vacuoles (Electron micrograph 5100X).

Small pieces from the cyst of case 1 were fixed in glutaraldehyde solution and processed for electron microscopic analysis. The cystic lumen was lined with cells showing multiple microvilli (Fig. 8) on the apical side. The cells under the epithelium (macrophages) had numerous intracellular vacuoles (Fig. 9).

## DISCUSSION

Exclusively thyroid-type cysts have seldom been reported. In recent literature, Szyfelbein, Young and Scully^2^ described a series of 20 cases, mostly revealed during consultations. The extreme rarity of the cystic variety of struma ovarii may make its identification difficult. Upon microscopic study, the quantity of thyroid follicles may be minimal, resulting in confusion with other cystic ovarian tumors.

Struma ovarii occurs in patients with a substantially higher average age than for those with common mature teratomas. They usually present a palpable abdominal mass and the tumors are unilateral and range from very small lesions up to as large as 10 cm in diameter. A clue to the diagnosis is the presence of a green to brown glairy fluid. The patients described in this report had the signs and symptoms of a unilateral adnexal mass and were 44 and 60 years old. The microscopic diagnosis of struma was found to be difficult for both cysts. In case 1, the diagnosis from frozen sections was benign and serous cystadenoma. In the 2nd case, frozen sections could not be made because of heavy calcification and the diagnosis was benign and probably mature cystic teratoma, based on the macroscopic appearance.

The final (paraffin) diagnosis in case 1 was reached following extensive sampling of the surgical specimen and special studies (immunohistochemistry). Thyroid follicles were present in only one out of 19 sections studied. The identity of this epithelium could be established using immunohistochemical stains. The ultrastructural patterns were similar to those of follicular cells. Therefore, the hypothesis of associated serous cystadenoma was rejected. The absence of any cilia was an initial clue that the cyst was not serous.

It is possible that several of the reported cases of struma associated with cystadenoma represented an exclusively thyroid-type cyst.^2^ Follicular epithelia of both normal and goitrous thyroids have been shown to react with anti-VIM in only a few cells.^3^ In case 2, the epithelial lining was extensively missing, the cystic wall heavily calcified, and typical thyroid follicles could be found in different areas of its wall. The classic features of dermoid cysts were not documented in any of the microscopic sections.

The frozen section technique is widely utilized in ovarian neoplasms, for which its usefulness is well accepted.^4^ Frozen sections of the tumors were correctly reported to be benign, but their precise nature was diagnosed erroneously in comparison to diagnosis using permanent sections. The inaccuracy in the frozen-section results was attributed to microscope sampling (case 1), and technical inability to cut calcified tissue (case 2). The tissue that could have led to the correct diagnosis being suspected in case 1 was contained in a portion of tissue sent for paraffin section but not for frozen section. In case 2, a benign diagnosis based on gross inspection alone was correct. The urgency of releasing intraoperative reports restricts the number of sections examined to below the ideal, especially when large specimens are submitted.^4^ Merely reporting a "benign" or "malignant" diagnosis may be enough to satisfy the surgeon's need to promote appropriate care for the patient. It will continue to be difficult to accurately diagnose cystic struma from frozen sections, because of the extensive sampling required and the inexperience of pathologists with this extremely rare tumor.

Cystic struma ovarii should be introduced into the differential diagnosis of all ovarian cysts with no macroscopic clue for the correct diagnosis. During intraoperative examination, careful gross evaluation is critical for the selection of tissue for frozen-section analysis, in view of the urgency of releasing reports and considering that the diagnostic areas are scarce. Special studies may be necessary to establish the appropriate diagnosis.
